# Availability and accessibility of monoclonal antibodies in Bosnia and Herzegovina: Findings and implications

**DOI:** 10.1177/23992026211027692

**Published:** 2021-07-09

**Authors:** Biljana Tubic, Vanda Marković-Peković, Saša Jungić, Eleonora Allocati, Brian Godman

**Affiliations:** 1 Agency for Medicinal Products and Medical Devices of Bosnia and Herzegovina, Banja Luka, Bosnia and Herzegovina; 2 Department of Medicinal Chemistry, University of Banja Luka, Banja Luka, Bosnia and Herzegovina; 3 Department of Social Pharmacy, University of Banja Luka, Banja Luka, Bosnia and Herzegovina; 4 University of Banja Luka, Banja Luka, Bosnia and Herzegovina; 5 University Clinical Centre of Republic of Srpska, Banja Luka, Bosnia and Herzegovina; 6 Istituto di Ricerche Farmacologiche ‘Mario Negri’ IRCCS, Milan, Italy; 7 Strathclyde Institute of Pharmacy and Biomedical Sciences, University of Strathclyde, Glasgow, UK; 8 Division of Public Health Pharmacy and Management, School of Pharmacy, Sefako Makgatho Health Sciences University, Pretoria, South Africa; 9 School of Pharmaceutical Sciences, University Sains Malaysia, Penang, Malaysia

**Keywords:** Monoclonal antibodies, biosimilars, regulatory approach, demand-side measures, Bosnia and Herzegovina, insulin glargine

## Abstract

**Background:**

Monoclonal antibodies (mAbs) represent the most numerous and significant group of biotherapeutics. While mAbs have undoubtedly improved treatment for many chronic diseases, including inflammatory diseases, they are typically expensive for health care systems and patients. Consequently, access to mAbs has been a problem for many patients especially among Central and Eastern European (CEE) countries. However, biosimilars can potentially help with costs, although there are concerns with their effectiveness and safety. This includes biosimilars for long-acting insulin analogues.

**Aim:**

Assess the availability and use of biological medicines, including biosimilars within Bosnia and Herzegovina (B&H).

**Methods:**

Assess the availability of mAbs via the current lists of approved and accessed mAbs versus those licenced in Europe and the United States and their utilisation, as well as specifically insulin glargine and its biosimilars, within B&H.

**Results:**

The availability of the mAbs in B&H appears satisfactory, which is encouraging. However, current usage is limited to a few mAbs which is a concern for subsequent patient care especially with limited use of biosimilars to address issues of affordability. We also see limited use of biosimilar insulin glargine.

**Conclusion:**

The limited use of mAbs including biosimilars needs to be addressed in B&H to improve the future care of patients within finite resources. We will monitor these developments.

## Introduction

Monoclonal antibodies (mAbs) represent the most numerous and most significant group of biotherapeutics, with biological medicines for disease areas, such as cancer and inflammatory diseases now dominating medicines expenditure.^1^ The importance of mAbs has grown in recent years as they offer treatment options for patients with chronic and often disabling conditions, including autoimmune diseases.^2–4^ However, mAbs are expensive limiting their prescribing among Central and Eastern European (CEE) countries, including patients with rheumatoid arthritis and inflammatory bowel disease, which needs addressing under solidarity principles.^5–7^ There are also considerable differences in the availability and use of new oncology medicines across Europe, enhanced by cost issues.^8,9^ A reduction in the prices of mAbs through biosimilars can result in appreciable savings as well as increasing the number of patients accessing these medicines especially where there are high co-payments, alternatively budget concerns.^5,10,11^ While price reductions for biosimilars versus pre-patent originator prices have often been limited, this is changing as seen for Humira^®^ (89% price reduction) in the Netherlands and its biosimilar in Denmark (83% price reduction) and the United Kingdom (75% price reduction).^10,12,13^ This provides hope for the future.

We are aware that regulatory approval for biosimilars across countries is different to the originator, and typically involves abridged non-clinical and clinical data.^14,15^ However, a lack of trust in biosimilars, coupled with limited government policies enhancing their use, including prescribing targets for new patients and switching, has reduced their prescribing in practice despite numerous publications demonstrating similar effectiveness and safety.^10,16–20^ Experience with generics in Bosnia and Herzegovina (B&H) and wider has shown that trust among all key stakeholders is essential for savings without compromising care.^21,22^ The same is true for biosimilars.^10^

Consequently, there is a need to document current availability and accessibility of mAbs, including biosimilars, and use the findings to suggest ways forward to improve future care within finite resources to provide direction across countries. The same applies to biosimilars for long-acting insulin analogues given the increasing use of long-acting insulin analogues to reduce rates of hypoglycaemia among insulin-dependent diabetic patients, which can account for up to 30% or more of patients with diabetes, and the increasing cost of care of diabetic patients.^23–27^ Consequently, the objectives for this study were to assess the availability and use of biological medicines, including biosimilars, within B&H and use the findings to provide future guidance to the authorities in B&H and wider.

## Methods

B&H consists of the two constitutive entities, the Republic of Srpska and the Federation of B&H.^22,28^ Each entity is competent for the health care on its territory, as well as the Brčko District of B&H.

The project consists of three elements, including retrospective pricing and utilisation analyses. The first element involved determining the current list of approved mAbs in B&H. The second element involved a comparison between the list of mAbs approved in B&H versus those actually reimbursed, including biosimilars as well as assessing current utilisation patterns. The last part involved retrospectively assessing utilisation patterns for long-acting insulin analogues versus total insulins, as well as utilisation patterns for biosimilar insulin glargine versus total insulin glargine as the first long-acting insulin analogue biosimilar available in B&H and across Europe.

A list of the approved mAbs for B&H market was created by interrogating the data base at the Agency for Medicinal Products and Medical Devices of B&H (ALMBIH), which is the regulatory authority at the state level,^29^ until early January 2021. This was undertaken by the principal co-authors (BT and VMP). While B&H is not a member of the EU, and does not apply the European Commission’s marketing authorisation regulations directly, the laws in B&H regarding marketing authorisation including biosimilars have been based on EU regulations, for example, Directive 2001/83/EC.

The availability of mAbs was determined by comparing the list of approved mAbs for B&H versus those listed by the European Medicines Agency (EMA)^30^ and the Food and Drug Administration (FDA), based on Lu *et al.* (2020), again by the principal co-authors (BT and VMP).^31,32^ Medicines were listed by their anatomical-therapeutic-chemical (ATC)^33^ classification to aid comparisons as there could be differences in the names of originators and biosimilars between countries. We included the United States to give a more complete picture as we are aware that a number of new biologics especially for oncology are given accelerated approval in the United States.^34^

Affordability in the first instance was assessed by comparing the list of medicines in ALMBIH with those reimbursed within the Health Insurance Fund of Republic of Srpska (HIF-RS), the Health Insurance and Reinsurance Institute of the Federation of B&H (HIRI-FB&H) and the Health Insurance Fund of the Brčko District of B&H (HIF-BD). Subsequently, measuring actual packs dispensed from 2017 to 2019 from the Health Insurance Fund data again via the principal co-authors (BT and VMP). The Health Insurance Fund data are robust and we have used these before in previous research projects.^22,35^ We chose packs dispensed as the use of defined daily doses (DDDs) is difficult in cancer due to typically multiple indications for oncology medicines.^35^ Wholesale prices for the different infliximab preparations were again taken from Health Insurance Fund data.

We also looked specifically at long-acting insulin analogues and their biosimilars, with long-acting insulin analogues typically appreciably more expensive than other forms of insulin.^36,37^ However, increasingly recognised patient benefits to reduce hypoglycaemia and enhance patient adherence has increased their use across countries, including developing countries,^27,36,38,39^ although this is not universal.^40^ In this situation, we will use DDDs to document utilisation patterns, similar to previous studies,^22^ and compare the findings with other countries.^27,39,40^

In accordance with local legislation neither approval from an ethics committee nor informed consent is required as this study did not deal directly with patients.

## Results

There were 96 mAbs approved by the FDA and EMA until early January 2021 (Table 1A in the Appendix). Seventy-six (79.2%) were approved jointly, 19 (19.8%) by the FDA and not by the EMA and 1 (1.04%) solely by EMA. However, several have been withdrawn. Perhaps not surprisingly given rising expenditures for oncology medicines in recent years combined with the high number of new oncology medicines being researched versus other disease areas,^8,41,42^ the greatest number of approved mAbs were for antineoplastic and immunomodulating agents (ATC–L). These accounted for 63.5% of all mAbs.

**Table 1.** Utilisation of different mAbs in B&H broken down by originator and biosimilar 2017–2019.

ATC Code	INN	Brand name	Pharmaceutical form	Dosage and quantity	2017	2018	2019
					Utilisation	Utilisation	Utilisation
L01XC02	Rituximab	MABTHERA	Concentrate for solution for infusion	100 mg/10 mL	1612	1261	723
			Solution for injection	120 mg/1 mL	13	443	NM
			Concentrate for solution for infusion	500 mg/50 mL	1778	1527	961
			Solution for subcutaneous injection	1400 mg/11.7 mL	NM	NM	1216
			Solution for subcutaneous injection	1600 mg/13.4 mL	NM	NM	3
		BLITZIMA	Concentrate for solution for injection/infusion	10 mg/1 mL	NA	NM	NM
		ACELLBIA	Concentrate for solution for infusion	10 mg/1 mL	NA	NA	NA
			Concentrate for solution for infusion	10 mg/1 mL	NA	NA	NA
		RIXATHON	Concentrate for solution for infusion	10 mg/1 mL (10 mL)	NA	NM	NM
			Concentrate for solution for infusion	10 mg/1 mL (2 × 10 mL)	NA	NM	NM
			Concentrate for solution for infusion	10 mg/1 mL (50 mL)	NA	NM	NM
			Concentrate for solution for infusion	10 mg/1 mL (2 × 50 mL)	NA	NM	NM
L01XC03	Trastuzumab	HERCEPTIN	Powder for concentrate for solution for infusion	150 mg	5969	5929	4568
		HERCEPTIN	Solution for injection	600 mg/5 mL	2810	3014	3885
		HERTICAD	Powder for concentrate for solution for infusion	150 mg	NA	NA	NA
		KANJINTI	Powder for concentrate for solution for infusion	150 mg	NA	NA	NA
		KANJINTI	Powder for concentrate for solution for infusion	420 mg	NA	NA	NA
		OGIVRI	Powder for concentrate for solution for infusion	150 mg	NA	NA	NM
		HERZUMA	Powder for concentrate for solution for infusion	420 mg	NA	NA	NA
		HERZUMA	Powder for concentrate for solution for infusion	150 mg	NA	NA	NA
L04AB02	Infliximab	REMICADE	Powder for concentrate for solution for infusion	100 mg	975	1180	1605
		REMSIMA	Powder for concentrate for solution for infusion	100 mg	420	450	520
		INFLECTRA	Powder for concentrate for solution for infusion	100 mg	671	81	74
L04AB04	Adalimumab	HUMIRA	Solution for injection	40 mg/0.8 mL	1152	320	NM
		HUMIRA	Solution for injection in pre-filled syringe	40 mg/0.4 mL	500	1899	2605
		AMGEVITA	Solution for injection in pre-filled syringe	40 mg/0.8 mL	NA	NA	20
		AMGEVITA	Solution for injection in pre-filled syringe	20 mg/0.4 mL	NA	NA	NM
		AMGEVITA	Solution for injection in pre-filled pen	40 mg/0.8 mL 2 pre-filed pens	NA	NA	NM
		HULIO	Solution for injection	40 mg/0.8 mL 1 pre-filled pen	NA	NA	NA
		HULIO	Solution for injection in pre-filled syringe	40 mg/0.8 mL 2 pre-filled syringes	NA	NA	NA
		HULIO	Solution for injection in pre-filled syringe	40 mg/0.8 mL 1 pre-filled syringe with 2 alcohol pads	NA	NA	NA
		HULIO	Solution for injection in pre-filled syringe	40 mg/0.8 mL 2 pre-filled syringes with 2 alcohol pads	NA	NA	NA

Shaded: biosimilar; NM: not marketed; NA: not approved.

There were 30 (31.25%) mAbs approved by ALMBIH by early January 2021 out of those approved by the EMA and FDA, with again most, that is, 22 (73.3%), for ATC-L group. These included the latest generation of oncology medicines, which are the checkpoint inhibitors, including pembrolizumab (L01XC18) and atezolizumab (L01XC32). Encouragingly, there appeared to be reasonably equal access to approved mAbs by the ALMBIH for all citizens in the different parts (entities) of B&H (Table 1A), with access to mAbs via HIF-RS and HIRI-FB&H only possible in well-defined therapeutic indications, typically in line with ALMBIH approval.

There were 22 mAbs accessible via HIF-RS (73.3%), although basiliximab is currently not approved by ALMBIH. Basiliximab is reimbursed for prophylaxis of acute organ rejection in *de novo* allogeneic renal transplantation in patients with panel reactive antibodies less than 80%, or in a triple maintenance immunosuppressive regimen containing cyclosporine for microemulsion, corticosteroids and either azathioprine or mycophenolate mofetil. Eighteen mAbs were accessible via HIRI-FB&H (60.0%), with currently not approved, but reimbursed nivolumab as monotherapy for advanced (unresectable or metastatic) melanoma.

The greatest number of reimbursed mAbs in B&H belong to the L01 group: 10 mAbs (33.3%) in the Republic of Srpska and 12 (40.0%) in the Federation of B&H (Table 2A). In the Brčko District of B&H, three mAbs are reimbursed, adalimumab, secukinumab and vedolizumab from ATC group L04.

**Table 2.** Wholesale prices of different infliximab presentations in B&H.

Name	Pharmaceutical form	Content concentration	Wholesale price per pack (EUR)		
			2017	2018	2019
REMSIMA	Powder for concentrate for solution for infusion	100 mg/1 vial	421.49	377.90	153.99
INFLECTRA	Powder for concentrate for solution for infusion	100 mg/1 vial	415.22	377.90	373.81
REMICADE	Powder for concentrate for solution for infusion	100 mg/1 vial	529.19	454.47	441.31

**Figure 1.**
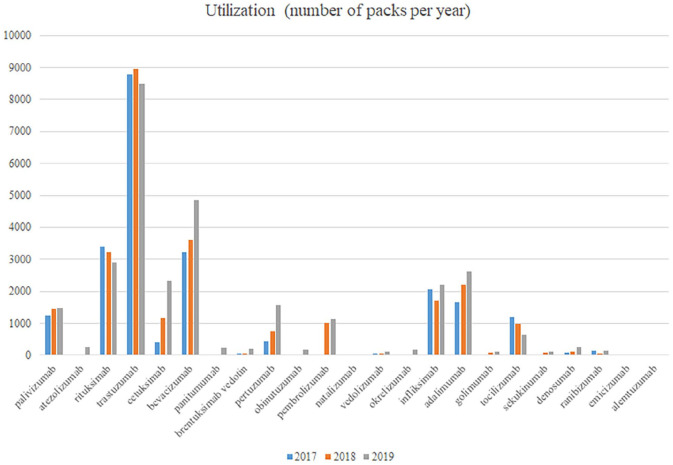
Utilisation (number of packs) of mAbs per year.

There are currently four approved biosimilars for mAbs in B&H, which are rituximab, trastuzumab, infliximab and adalimumab (Table 2A), with the greatest number of approvals for trastuzumab biosimilars. This may have facilitated their use; however, there appears only limited changes in utilisation patterns for mAbs between 2017, with biosimilars for infliximab launched in 2016, and 2019 (Figure 1) with currently limited use of biosimilars in recent years despite being marketed (Table 1). The exception is bevacizumab, which is currently unavailable as a biosimilar. Limited use of the other mAbs may well reflect issues of affordability despite being listed on the reimbursement lists of the different entities of B&H.

The limited use of biosimilar infliximab is despite an appreciable fall in prices versus the originator in recent years (Table 2).

We have seen increasing use of long-acting insulins in B&H in recent years rising from 19.6% of total insulin utilisation in 2014 to 35.5% in 2019, with expenditure increasing to 45.1% of total insulin expenditure in 2019, reflecting perceived patient benefits despite increasing costs. Overall costs could have been reduced with the availability of biosimilars. However, this was hampered by only limited differences in prices between the originator and biosimilar insulin glargine 100 IU/ml at 6.8% and 7.9% in 2018 and 2019, respectively. In addition, high use of the patented 300 IU/ml formulation at 52.1% of total insulin glargine in 2019 as a result of promotional activities by the company. Overall, limited use of biosimilar insulin glargine at only 6.17% of total insulin glargine 100 IU/ml in 2019. This again reflects limited demand-side measures instigated by the authorities in B&H to counter-act the activities of the originator company. We have seen a similar situation in a number of other CEE countries, including Estonia, Latvia and Romania resulting in limited or no use of biosimilar insulin glargine.^39^

## Discussion

Encouragingly, there was reasonable listing of the mAbs among the various entities in B&H given concerns generally with the availability and reimbursement of biologic medicines among CEE countries.^5,6^ In addition, reasonable usage of medicines for patients with cancer, including rituximab and trastuzumab, and those with immune diseases, such as rheumatoid arthritis, including infliximab and adalimumab. However, limited use of the majority of mAbs (Figure 1) suggests issues with available funding despite being listed on the reimbursement lists in B&H. This is a concern when seeking to improve patient care in these patients. It may be that increased availability of biosimilars at considerably lower prices could help along with increased physician and patient education regarding the regulatory approaches for biosimilars and studies demonstrating similar effectiveness and safety with originators.^10,17,20^ This builds on examples in other European countries where there have been considerable use of biosimilars and corresponding savings following multiple demand-side measures^10,18,43^ as well as a number of countries with biosimilars of insulin glargine.^27,39^

However, physicians and health authorities need to instigate policies to enhance the use of biosimilars in B&H building on successful experiences in other countries.^18,44^ These include educational policies to address concerns and lack of trust with biosimilars given the impact of the nocebo effect in this area,^44,45^ alongside prescribing targets and restrictions for more expensive originators.^10,43,44^ Otherwise, there will continue to be limited use of biosimilars.^16^ This is a concern given the potential for appreciable savings with biosimilars as seen with biosimilar infliximab in B&H (Table 2) without compromising care.^17^

Lack of trust and use of biosimilars in B&H may be hampered by issues, such as interchangeability and substitutability, with these issues currently not being clearly defined by the ALMBIH. Consequently, there is a need for B&H to learn from other European countries to instigate appropriate educational and other measures to appreciably increase biosimilar use to benefit patients especially given current budgetary issues and competing demands under opportunity cost considerations.^10,18,43,46^ Increased competition can lower prices of both originator mAbs and biosimilars as seen recently with adalimumab in a number of European markets.^12,18^ Such approaches may assist in the Brčko District where infliximab is currently not on the list of reimbursed medicines. Lower prices of biosimilars building on existing reductions (Table 2), along with greater patient and physician trust, should enhance their availability and use for the benefit of patients. We will be investigating this further especially with ALMBIH increasingly encouraging physicians to prescribe biosimilars, which should enhance the attractiveness of the biosimilar market and address current concerns with their lack of availability and use (Tables 1 and 2A).

We are aware of a number of limitations with this study. These include the fact that we only included data for 3 years for the mAbs. In addition, we did not contact physicians directly to ascertain the rationale behind the utilisation patterns seen. Despite these limitations, we believe our findings are robust providing direction for the future.

## Conclusion

In conclusion, there appeared to be good availability of mAbs in B&H. However, there is currently limited use of a number of these due to issues of affordability, and we also see limited use of biosimilars, including biosimilar insulin glargine. Both can be addressed by enhancing the attractiveness of the market for biosimilars, benefitting all key stakeholder groups. B&H can learn from other European countries.

## Supplemental Material

sj-pdf-1-map-10.1177_23992026211027692 – Supplemental material for Availability and accessibility of monoclonal antibodies in Bosnia and Herzegovina: Findings and implications Click here for additional data file. Supplemental material, sj-pdf-1-map-10.1177_23992026211027692 for Availability and accessibility of monoclonal antibodies in Bosnia and Herzegovina: Findings and implications by Biljana Tubic, Vanda Marković-Peković, Saša Jungić, Eleonora Allocati and Brian Godman in Medicine Access @ Point of Care
