# Supplementary material for: Availability and accessibility of monoclonal antibodies in Bosnia and Herzegovina: Findings and implications
Source: Med Access Point Care. 2021 Jul 9;5:23992026211027692. doi: 10.1177/23992026211027692 (PMC9413607; doi:10.1177/23992026211027692)
Supplement: sj-pdf-1-map-10.1177_23992026211027692 – Supplemental material for Availability and accessibility of monoclonal antibodies in Bosnia and Herzegovina: Findings and implications [file sj-pdf-1-map-10.1177_23992026211027692.pdf]

Appendix

Table 1A: List of ALMBIH approved mAbs *versus* list of EMA/FDA approved mAbs and accessibility of the approved mAbs within the B&H healthcare funds

	**ATC**	**INN**	**mAbs (number of approvals issued by ALMBIH)**	**Biosimilars of the mAbs (number of approvals issued by ALMBIH)**	**mAbs accessible *via* HIFRS**	**mAbs accessible *via* HIRI F&B**	**Approval status of mAbs by EMA/FDA (updated by 14.01.2021)**
1.	B01AC13	abciximab	not approved	not approved	x	x	FDA approved
2.	B01AX07	caplacizumab	not approved	not approved	x	x	EMA/FDA approved
3.	B02BX06	emicizumab	4	not approved	√	x	EMA/FDA approved
4.	B06AC05	lanadelumab	not approved	not approved	x	x	EMA/FDA approved
5.	B06AX01	crizanlizumab	not approved	not approved	x	x	EMA/FDA approved
6.	C10AX13	evolocumab	not approved	not approved	x	x	EMA/FDA approved
7.	C10AX14	alirocumab	2	not approved	x	x	EMA/FDA approved
8.	D11AH05	dupilumab	not approved	not approved	x	x	EMA/FDA approved
9.	J05AX23	ibalizumab	not approved	not approved	x	x	EMA/FDA approved

10.	J06BB16	palivizumab	1	not approved	√	√	EMA/FDA approved
11.	J06BB18	raxibacumab	not approved	not approved	x	x	FDA approved
12.	J06BB21	bezlotoxumab	not approved	not approved	x	x	EMA/FDA approved
13.	J06BB22	obiltoxaximab	not approved	not approved	x	x	EMA/FDA approved
14.	L01XC02	rituximab	11	7	√	√	EMA/FDA approved
15.	L01XC03	trastuzumab	8	6	√	√	EMA/FDA approved
16.	L01XC05	gemtuzumab ozogamicin	not approved	not approved	x	x	EMA/FDA approved
17.	L01XC06	cetuximab	1	not approved	√	√	EMA/FDA approved
18.	L01XC07	bevacizumab	2	not approved	√	√	EMA/FDA approved
19.	L01XC08	panitumumab	1	not approved	x	√	EMA/FDA approved
20.	L01XC10	ofatumumab	not approved	not approved	x	x	FDA approved
21.	L01XC11	Ipilimumab	not approved	not approved	x	x	EMA/FDA approved
22.	L01XC12	brentuximab vedotin	1	not approved	√	√	EMA/FDA approved
23.	L01XC13	pertuzumab	1	not approved	√	√	EMA/FDA approved
24.	L01XC14	trastuzumab emtanzin	2	not approved	√	√	EMA/FDA approved
25.	L01XC15	obinutuzumab	1	not approved	√	√	EMA/FDA approved
26.	L01XC16	dinutuximab beta	not approved	not approved	x	x	EMA/FDA approved
27.	L01XC17	Nivolumab	not approved	not approved	x	√	EMA/FDA approved

28.	L01XC18	Pembrolizumab	2	not approved	√	√	EMA/FDA approved
29.	L01XC19	blinatumomab	not approved	not approved	x	x	EMA/FDA approved
30.	L01XC21	ramucirumab	not approved	not approved	x	x	EMA/FDA approved
31.	L01XC22	necitumumab	not approved	not approved	x	x	EMA/FDA approved
32.	L01XC23	elotuzumab	not approved	not approved	x	x	EMA/FDA approved
33.	L01XC24	daratumumab	2	not approved	x	x	EMA/FDA approved
34.	L01XC25	mogamulizumab	not approved	not approved	x	x	EMA/FDA approved
35.	L01XC26	inotuzumab ozogamicin	not approved	not approved	x	x	EMA/FDA approved
36.	L01XC27	olaratumab	not approved	not approved	x	x	FDA approved
37.	L01XC28	Durvalumab	not approved	not approved	x	x	EMA/FDA approved
38.	L01XC31	Avelumab	not approved	not approved	x	x	EMA/FDA approved
39.	L01XC32	Atezolizumab	2	not approved	√	√	EMA/FDA approved
40.	L01XC33	Cemiplimab	not approved	not approved	x	x	EMA/FDA approved
41.	L01XC34	moxetumomab pasudotox	not approved	not approved	x	x	FDA approved
42.	L01XC35	tafasitamab	not approved	not approved	x	x	FDA approved
43.	L01XC37	polatuzumab vedotin	not approved	not approved	x	x	EMA/FDA approved
44.	L01XC38	isatuximab	not approved	not approved	x	x	FDA/EMA approved

45.	L01XC39	belantamab mafodotin	not approved	not approved	x	x	FDA/EMA approved
46.	L04AA02	muromonab	not approved	not approved	x	x	FDA approved
47.	L04AA21	efalizumab	not approved	not approved	x	x	FDA approved
48.	L04AA23	natalizumab	1	not approved	√	√	EMA/FDA approved
49.	L04AA25	eculizumab	not approved	not approved	x	x	EMA/FDA approved
50.	L04AA26	belimumab	not approved	not approved	x	x	EMA/FDA approved
51.	L04AA33	vedolizumab	1	not approved	√	√	EMA/FDA approved
52.	L04AA34	alemtuzumab	1	not approved	x	x	EMA/FDA approved
53.	L04AA36	ocrelizumab	1	not approved	√	√	EMA/FDA approved
54.	L04AA39	emapalumab	not approved	not approved	x	x	FDA approved
55.	L04AA43	ravulizumab	not approved	not approved	x	x	EMA/FDA approved
56.	L04AA47	inebilizumab	not approved	not approved	x	x	FDA approved
57.	L04AB02	infliximab	3	2	√	√	EMA/FDA approved
58.	L04AB04	adalimumab	11	7	√	√	EMA/FDA approved
59.	L04AB05	certolizumab pegol	not approved	not approved	x	x	EMA/FDA approved
60.	L04AB06	golimumab	2	not approved	√	x	EMA/FDA approved
61.	L04AC01	daclizumab	not approved	not approved	x	x	FDA approved
62.	L04AC02	basiliximab	not approved	not approved	√	x	EMA/FDA approved
63.	L04AC05	ustekinumab	not approved	not approved	x	x	EMA/FDA approved
64.	L04AC07	tocilizumab	3	not approved	√	x	EMA/FDA approved

65.	L04AC08	canakinumab	not approved	not approved	x	x	EMA/FDA approved
66.	L04AC10	secukinumab	2	not approved	√	x	EMA/FDA approved
67.	L04AC11	siltuximab	not approved	not approved	x	x	EMA/FDA approved
68.	L04AC12	brodalumab	not approved	not approved	x	x	EMA/FDA approved
69.	L04AC13	ixekizumab	not approved	not approved	x	x	EMA/FDA approved
70.	L04AC14	sarilumab	not approved	not approved	x	x	EMA/FDA approved
71.	L04AC16	guselkumab	1	not approved	x	x	EMA/FDA approved
72.	L04AC17	tildrakizumab	not approved	not approved	x	x	EMA/FDA approved
73.	L04AC18	risankizumab	1	not approved	x	x	EMA/FDA approved
74.	L04AC19	satralizumab	not approved	not approved	x	x	FDA approved
75.	M05BX04	denosumab	1	not approved	x	x	EMA/FDA approved
76.	M05BX05	burosumab	not approved	not approved	x	x	EMA/FDA approved
77.	M05BX06	romosozumab	not approved	not approved	x	x	EMA/FDA approved
78.	N02CD01	erenumab	not approved	not approved	x	x	EMA/FDA approved
79.	N02CD02	galcanezumab	not approved	not approved	x	x	EMA/FDA approved
80.	N02CD03	fremanezumab	not approved	not approved	x	x	EMA/FDA approved
81.	R03DX05	omalizumab	2	not approved	x	x	EMA/FDA approved
82.	R03DX08	reslizumab	not approved	not approved	x	x	EMA/FDA approved
83.	R03DX09	mepolizumab	not approved	not approved	x	x	EMA/FDA approved
84.	R03DX10	benralizumab	not approved	not approved	x	x	EMA/FDA approved
85.	S01LA04	ranibizumab	1	not approved	√	x	EMA/FDA approved

86.	S01LA06	brolucizumab	not approved	not approved	x	x	EMA/FDA approved
87.	V03AB37	idarucizumab	1	not approved	x	x	EMA/FDA approved
88.	V09HA03	Besilesomab	not approved	not approved	x	x	EMA approved
89.	V10XX02	ibritumomab tiuxetan (90Y)	not approved	not approved	x	x	EMA/FDA approved
90.	NONE	teprotumumab	not approved	not approved	x	x	FDA approved
91.	NONE	eptinezumab	not approved	not approved	x	x	FDA approved
92.	NONE	sacituzumab	not approved	not approved	x	x	FDA approved
93.	NONE	atoltivimab, maftivimab, and odesivimab	not approved	not approved	x	x	FDA approved
94.	NONE	naxitamab	not approved	not approved	x	x	FDA approved
95.	NONE	margetuximab	not approved	not approved	x	x	FDA approved
96.	NONE	ansuvimab	not approved	not approved	x	x	FDA approved

NB: HIF RS – The Health Insurance Fund of Republic of Srpska; HIRI F&B - the Health Insurance and Reinsurance Institute of the Federation of B&H; ATC – Anatomical-Therapeutic Classification; INN – International Nonproprietary Name; mAbs – monoclonal antibodies; ALMBIH Agency for Medicines and Medical Devices of Bosnia and Herzegovina; x-not accessed; √ accessed

Table 2A: Approved biosimilars for B & H market

ATC code	INN	Invented (Brand) name	Pharmaceutical Form	Content concentration	Pack size	Manufacturer	Approval date
L01XC02	rituximab	BLITZIMA	Concentrate for Solution for Injection/Infusion	10 mg/1 mL	1 Bottle (50 mL)	CELLTRION Inc.	25.09.2018
		ACELLBIA	Concentrate for Solution for Infusion	10 mg/1 mL	1 Bottle (50 mL)	JSC BIOCAD	25.03.2020
			Concentrate for Solution for Infusion	10 mg/1 mL	2 Bottles per 10 mL		
		RIXATHON	Concentrate for Solution for Infusion	10 mg/1 mL	1 Bottle (10 mL)	LEK farmacevtska družba d.d.	27.09.2019
			Concentrate for Solution for Infusion	10 mg/1 mL	2 Bottles per 10 mL		
			Concentrate for Solution for Infusion	10 mg/1 mL	1 Bottle (50 mL)		
			Concentrate for Solution for Infusion	10 mg/1 mL	2 Bottles per 50 mL		
L01XC03	trastuzumab	HERTICAD	Powder for concentrate for solution for infusion	150 mg	1 Bottle (150 mg)	JSC BIOCAD	25.03.2020
		KANJINTI	Powder for concentrate for solution for infusion	150 mg	1 Bottle (150 mg)	AMGEN (Europe) GmbH	23.01.2020
			Powder for concentrate for solution for infusion	420 mg	1 Bottle (420 mg)		

		OGIVRI	Powder for concentrate for solution for infusion	150 mg	1 Bottle (150 mg)	BGP PRODUCTS OPERATIONS GmbH	26.07.2019
		HERZUMA	Powder for concentrate for solution for infusion	420 mg	1 Bottle (420 mg)	CELLTRION Inc.	25.12.2020
			Powder for concentrate for solution for infusion	150 mg	1 Bottle (150 mg)		
L04AB02	infliximab	INFLECTRA	Powder for concentrate for solution for infusion	100 mg	1 Bottle (100 mg)	PFIZER LUXEMBOURG SARL	02.03.2020
		REMSIMA	Powder for concentrate for solution for infusion	100 mg	1 Bottle (100 mg)	CELLTRION Inc.	13.05.2020
L04AB04	adalimumab	AMGEVITA	solution for injection in pre-filled syringe	40 mg/0.8 mL	2 pre-filled syringe per 0.8 mL solution for injection	AMGEN (Europe) GmbH	13.08.2019
			solution for injection in pre-filled syringe	20 mg/0.4 mL	1 pre-filled syringe per 0.4 mL solution for injection		
			solution for injection in pre-filled pen	40 mg/0.8 mL	2 pre-filled pen per 0.8 mL solution for injection		
		HULIO	solution for injection	40 mg/0.8 mL	1 pre-filled pen	BGP PRODUCTS OPERATIONS GmbH	29.10.2020
			solution for injection in pre-filled syringe	40 mg/0.8 mL	2 pre-filled syringes		
			solution for injection in pre-filled syringe	40 mg/0.8 mL	1 pre-filled syringe with 2 alcohol pads		
			solution for injection in pre-filled syringe	40 mg/0.8 mL	2 pre-filled syringes with 2 alcohol pads		
